# Thermodynamic Analysis of Time Evolving Networks

**DOI:** 10.3390/e20100759

**Published:** 2018-10-02

**Authors:** Cheng Ye, Richard C. Wilson, Luca Rossi, Andrea Torsello, Edwin R. Hancock

**Affiliations:** 1 Department of Computer Science, Royal Holloway, University of London, Egham TW20 0EX, UK; 2 Department of Computer Science, University of York, York YO10 5GH, UK; 3 School of Engineering and Applied Science, Aston University, Birmingham B4 7ET, UK; 4 Dipartimento di Scienze Ambientali, Informatica, Statistica Universita’ Ca’ Foscari Venezia via Torino 155, 30172 Venezia Mestre, Italy; 5 Beijing Advanced Innovation Center for Big Data and Brain Computing, Beihang University, Beijing 100083, China

**Keywords:** time-varying complex networks, von Neumann entropy, internal energy, temperature

## Abstract

The problem of how to represent networks, and from this representation, derive succinct characterizations of network structure and in particular how this structure evolves with time, is of central importance in complex network analysis. This paper tackles the problem by proposing a thermodynamic framework to represent the structure of time-varying complex networks. More importantly, such a framework provides a powerful tool for better understanding the network time evolution. Specifically, the method uses a recently-developed approximation of the network von Neumann entropy and interprets it as the thermodynamic entropy for networks. With an appropriately-defined internal energy in hand, the temperature between networks at consecutive time points can be readily derived, which is computed as the ratio of change of entropy and change in energy. It is critical to emphasize that one of the main advantages of the proposed method is that all these thermodynamic variables can be computed in terms of simple network statistics, such as network size and degree statistics. To demonstrate the usefulness of the thermodynamic framework, the paper uses real-world network data, which are extracted from time-evolving complex systems in the financial and biological domains. The experimental results successfully illustrate that critical events, including abrupt changes and distinct periods in the evolution of complex networks, can be effectively characterized.

## 1. Introduction

There has been a vast amount of effort expended on the problems of how to represent networks, and from this representation, derive succinct characterizations of network structure and in particular how this structure evolves with time [1,2,3]. Broadly speaking, the representations and the resulting characterizations are goal-directed and have centred on ways of capturing network substructure using clusters or notions such as hubs and communities [4,5,6,7]. Here, the underlying representations are based on the connectivity structure of the network or statistics that capture the connectivity structure such as degree distributions [2,8,9].

A more principled approach is to try to characterize the properties of networks using ideas from statistical physics [10,11]. Here, the network can be succinctly described using a partition function, and thermodynamic characterizations of the network such as entropy [12], total energy and temperature can be derived from the partition function [13,14,15]. Specifically, statistical thermodynamics can be combined with both graph theory and kinetics to provide a practical framework for handling highly structured and highly interactive time-evolving complex systems [13]. By using a random walk that maximizes the Ruelle–Bowen free-energy rate on weighted graphs, a novel centrality measure can be computed, and this has been successfully applied to both connected and disconnected large-scale networks [14]. Recently, it has been demonstrated that the subgraph centrality can be interpreted as a partition function of a network [16], and as a result the entropy, internal energy and the Helmholtz free energy can be defined using spectral graph theory. The authors have also argued that the thermodynamic quantities are intimately related to the complex network dynamics. This approach combines the theoretical tools developed for studying graph spectra in the context of statistical mechanics of complex networks and clearly points out the potentials of the current approach to study real-world time-varying networks.

More recently, Minello et al. [17] have presented a quantum thermodynamic approach to study time-varying networks, in which the thermodynamic variables are developed through an unknown Hamiltonian operator governing the free evolution through the Schrödinger equation. Here, motivated by our recent work [18], we adopt a different theoretical foundation, namely the statistical mechanics, to establish our thermodynamic framework to analyse the time evolution of dynamic networks. We commence by studying undirected networks, and we define the network thermodynamic entropy, based on a recently-developed approximation for the von Neumann entropy. We then focus on developing additional thermodynamic variables, i.e., internal energy and temperature for time-varying networks. We further show that our framework can be readily applied to directed networks, by taking into consideration the difference between the in- and out-degree of network nodes. We evaluate the usefulness of the proposed method using real-world time-varying complex system data from both financial and biological domains.

### Related Literature

Although the bulk of existing network theory is concerned with static networks, most realistic networks are in reality dynamic in nature. Generally speaking, most existing methods for analysing the time evolution of complex networks have centred on studying structural measures of static networks and then applying these quantities to each snapshot of the time-varying network in order to understand the evolutionary patterns. For instance, Holme et al. [19] have analysed the time evolution of a number of well-known network features, including clustering coefficient, degree-degree correlations, average geodesic length and reciprocity of a large-scale online social network. Moreover, in [20], the authors have analysed how the social networks of Flickr and Yahoo!360 evolve over different time periods using measures such as network density and average distance between nodes in the network components. Although such methods have proven to be efficient in reflecting the time evolution of some structural properties of evolving networks, they have a significant drawback, namely the lack of the use of structure information between temporal networks at two consecutive time steps, e.g., the node degree change and edge number change.

In order to overcome this problem and to incorporate the missing structure information, a number of alternative techniques to capture the structure and evolution of networks have been proposed. For instance, Palla et al. [21] have developed a method for investigating the time dependence of the overlapping communities on a social network, using the clique percolation method. Specifically, they take into consideration both the group size and age and propose a measure for quantifying the relative overlap between two states of the same community at different time steps. Furthermore, they have developed a new network indicator called stationarity in order to quantify the changing rate of communities based on their size and age. In this way, the authors have managed to exploit the community structure information between subsequent states of a time-evolving network. More recently, Peel and Clauset [22] have formalized the problem of identifying change points during network evolution within an online probabilistic learning framework and have utilized generative network models and statistical hypothesis tests to solve it. This method has proven to detect successfully if, when and how change points occur in two high-resolution dynamic social networks.

Compared to the existing evolving network analysis approaches, our thermodynamics analysis provides an advantageous approach in that the thermodynamic quantities, especially the temperature, fully exploit the information related to the structural changes of networks at subsequent time steps. More importantly, our approach does not require computationally complicated Bayesian probabilistic frameworks such as the generalized hierarchical random graph (GHRG) model [22], but only uses a number of simple, but important network characteristics, i.e., node degree statistics, edge number and degree information of some simple substructures such as triangles. This yields a low computational complexity to our thermodynamic analysis.

The structure of the remainder of the paper is as follows. Section 2 gives a detailed development of the thermodynamic framework for network evolution analysis. Section 3 tests the proposed method on a number of real-world time-varying networks, i.e., the New York Stock Exchange (NYSE) network and fruit fly life cycle gene expression network. Section 4 summarizes the main contributions of this paper and also suggests a few research directions for the future.

## 2. Thermodynamic Framework for Time-Evolving Complex Networks

In this section, we provide a detailed development of the thermodynamic framework for analysing the time evolution of complex networks. In particular, the framework consists of three thermodynamic variables, namely the thermodynamic entropy, internal energy and temperature. Mathematically, the thermodynamic entropy takes the same form as the network von Neumann entropy, when we associate the microscopic configurations of a network with the eigenstates of the normalized Laplacian spectrum. By defining an appropriate internal energy, the temperature is determined by measuring fluctuations in entropy and internal energy. We show that computationally, the framework is effective since each of these thermodynamic variables can be calculated using a few important graph statistics including number of nodes and edges and node degree statistics.

### 2.1. Initial Considerations

Let G(V,E) be an undirected graph with node set *V* and edge set E⊆V×V. The adjacency matrix *A* of graph G(V,E) is defined as:

(1) $$A_{uv}=\left\{\begin{array}{cc} 1 & \mathrm{if}\;(u,v)\in E\\ 0 & \mathrm{otherwise}. \end{array}\right.$$

The degree of node *u* is $d_{u}=\sum_{v\in V}A_{vu}.$ The normalized Laplacian matrix is $\tilde{L}=D^{-1/2}\left(D-A\right)D^{-1/2}$, where *D* is the degree diagonal matrix whose elements are given by $D_{uu}=d_{u}$ and zeros elsewhere. The element-wise expression of $\tilde{L}$ is:

(2) $${\tilde{L}}_{uv}=\left\{\begin{array}{cc} 1 & \mathrm{if}\;u=v\;\mathrm{and}\;d_{v}\neq 0\\ -\frac{1}{\sqrt{d_{u}d_{v}}} & \mathrm{if}\;u\neq v\;\mathrm{and}\;(u,v)\in E\\ 0 & \mathrm{otherwise}. \end{array}\right.$$

The normalized Laplacian matrix $\tilde{L}$ and its spectrum yield a number of very useful graph invariants for a finite graph. For example, the eigenvalues for the graph normalized Laplacian are real numbers, bounded between zero and two [23].

According to [24], the normalized Laplacian matrix $\tilde{L}$ can be interpreted as the density matrix of an undirected graph. With this choice of density matrix, the von Neumann entropy of the undirected graph is defined as the Shannon entropy associated with the normalized Laplacian eigenvalues, i.e.,

(3) $$H_{VN}=-\sum_{i=1}^{\left|V\right|}\frac{{\tilde{\lambda }}_{i}}{\left|V\right|}\ln\frac{{\tilde{\lambda }}_{i}}{\left|V\right|}$$

where $\tilde{\lambda_{i}}$, i=1,…,|V|, are the eigenvalues of $\tilde{L}$.

In this paper, we aim at developing a thermodynamic characterization of network structure. We commence by assuming that at any instant in time, a network G(V,E) is statistically distributed across an ensemble of |V| microstates. The probability that the system occupies a microstate indexed *s* is given by $p_{s}={\tilde{\lambda }}_{s}/\sum_{s=1}^{\left|V\right|}{\tilde{\lambda }}_{s},$ where ${\tilde{\lambda }}_{s},s=1,2,\ldots ,\left|V\right|$ are the eigenvalues of the normalized Laplacian matrix of graph *G*. Noting that the trace of a matrix is the sum of its eigenvalues, we have $\sum_{s=1}^{\left|V\right|}{\tilde{\lambda }}_{s}=Tr\left[\tilde{L}\right]=\left|V\right|,$ so the microstate occupation probability is simply $p_{s}={\tilde{\lambda }}_{s}/\left|V\right|.$

We define the thermodynamic entropy of a network using the Shannon formula, which is exclusively dependent on the probabilities of the microstates:

(4) $$H_{S}=-k\sum_{s=1}^{\left|V\right|}p_{s}\ln p_{s}=-k\sum_{s=1}^{\left|V\right|}\frac{{\tilde{\lambda }}_{s}}{\left|V\right|}\ln\frac{{\tilde{\lambda }}_{s}}{\left|V\right|},$$

where *k* is the Boltzmann constant and is set to be one to simplify matters.

It is clear that the thermodynamic entropy Equation (4) and the von Neumann entropy Equation (3) take the same form. Both depend on the graph size and the eigenvalues of the normalized Laplacian matrix. It is reasonable to suggest that the von Neumann entropy can be interpreted as the thermodynamic entropy of a complex network.

### 2.2. Approximate von Neumann Entropy for Undirected Graphs

In prior work [25], we have shown how the von Neumann entropy of an undirected graph Equation (3) can be simplified by making use of the quadratic approximation (i.e., −xlnx≈x(1−x)),

(5) $$H_{Q}=\sum_{i=1}^{\left|V\right|}\frac{{\tilde{\lambda }}_{i}}{\left|V\right|}\left(1-\frac{{\tilde{\lambda }}_{i}}{\left|V\right|}\right).$$

For undirected graphs, this quadratic approximation allows the von Neumann entropy to be expressed in terms of the trace of the normalized Laplacian and the trace of the squared normalized Laplacian, with the result that:

(6) $$H_{VN}=\frac{Tr[\tilde{L}]}{\left|V\right|}-\frac{Tr[{\tilde{L}}^{2}]}{{\left|V\right|}^{2}}.$$

The two traces appearing in the above expression are given in terms of node degree statistics [25], leading to:

(7) $$H_{VN}=1-\frac{1}{\left|V\right|}-\frac{1}{{\left|V\right|}^{2}}\sum_{(u,v)\in E}\frac{1}{d_{u}d_{v}}.$$

This formula contains two measures of graph structure: the first is the number of nodes of the graph, while the second is based on degree statistics for pairs of nodes connected by edges. Moreover, the expression for the approximate entropy has computational complexity that is quadratic in graph size, which is simpler than the original von Neumann entropy that is cubic, since it requires enumeration of the normalized Laplacian spectrum.

In order to obtain a better understanding of the entropic measure of graphs, it is interesting to explore how the von Neumann entropy is bounded for graphs of a particular size, and in particular which topologies give the maximum and minimum entropies. From Equation (7), it is clear that when the term under the summation is minimal, the von Neumann entropy reaches its maximal value. This occurs when each pair of graph nodes is connected by an edge, and this means that the graph is complete. On the other hand, when the summation takes on its maximal value, the von Neumann entropy is minimum. This occurs when the structure is a string.

The maximum and minimum entropies corresponding to these cases are as follows. For a complete graph $K_{n}$, in which each node has degree n−1, it is straightforward to show that:

$$H_{VN}\left(K_{n}\right)=1-\frac{1}{n}-\frac{1}{n^{2}}\cdot \frac{n(n-1)}{2{\left(n-1\right)}^{2}}=1-\frac{2n-1}{2n(n-1)}.$$

In the case of a string $P_{n}$ (n≥3), in which two terminal nodes have degree one, while the remainder have degree two, we have:

$$H_{VN}\left(P_{n}\right)=1-\frac{1}{n}-\frac{1}{n^{2}}\cdot \frac{n+1}{4}=1-\frac{5n+1}{4n^{2}}.$$

As a result, the graph von Neumann entropy is bounded as follows:

$$1-\frac{5|V|+1}{{4|V|}^{2}}\leq H_{VN}\left(G\right)\leq 1-\frac{2|V|-1}{2|V|(|V|-1)}$$

where the lower boundary is obtained for strings, which are the simplest regular graph, and the upper bound is reached for complete graphs.

### 2.3. Internal Energy and Temperature

The internal energy of a network is defined as the mean value of the total energy, i.e., the sum of all microstate energies, each weighted by its occupation probability:

(8) $$U=\sum_{s=1}^{\left|V\right|}p_{s}U_{s},$$

where $U_{s}$ is the energy of microstate *s*. Here, we take the internal energy to be the total number of edges in the graph i.e., U=|E|. From the properties of the Laplacian and normalized Laplacian matrices, we have $\left|E\right|=Tr\left[L\right]=Tr\left[D^{1/2}\tilde{L}D^{1/2}\right]=Tr\left[D\tilde{L}\right]$. This can be achieved if we set the microstate energies to be $U_{s}=\left|V\right|d_{s}$, i.e., proportional to the node degrees.

Suppose that the graphs G=(V,E) and $G^{\prime }=\left(V^{\prime },E^{\prime }\right)$ represent the structure of a time-varying complex network at two consecutive epochs *t* and $t^{\prime }$, respectively. The reciprocal of the thermodynamic temperature *T* is the rate of change of entropy with internal energy, subject to the condition that the volume and number of particles are held constant, i.e., $1/T=dH_{VN}/dU.$ This definition can be applied to evolving complex networks, which do not change size during their evolution.

#### 2.3.1. Undirected Edges

We approximate the change of the von Neumann entropy $H_{VN}$ between undirected graphs *G* and $G^{\prime }$ as:

$$dH_{VN}=H_{VN}\left(G^{\prime }\right)-H_{VN}\left(G\right)=\sum_{\left(u,v\right)\in E,E^{\prime }}\frac{d_{u}\Delta_{v}+d_{v}\Delta_{u}+\Delta_{u}\Delta_{v}}{d_{u}\left(d_{u}+\Delta_{u}\right)d_{v}\left(d_{v}+\Delta_{v}\right)},$$

where $\Delta_{u}$ is the change of the degree of node *u*: $\Delta_{u}=d_{u}^{\prime }-d_{u}$; $\Delta_{v}$ is similarly defined. The change in internal energy is equal to the change in the total number of edges: $dU=U\left(G^{\prime }\right)-U\left(G\right)=|E^{\prime }\left|-|E\right|=\Delta |E|.$ Hence, the reciprocal temperature *T* is:

(9) $$\frac{1}{T(G,G^{\prime })}=\sum_{\left(u,v\right)\in E,E^{\prime }}\frac{d_{u}\Delta_{v}+d_{v}\Delta_{u}+\Delta_{u}\Delta_{v}}{\Delta |E|d_{u}\left(d_{u}+\Delta_{u}\right)d_{v}\left(d_{v}+\Delta_{v}\right)}.$$

When the changes in node degree are small compared to the node degree, i.e., $|\Delta_{u}|<<d_{u}$, then:

(10) $$\frac{1}{T(G,G^{\prime })}=\sum_{\left(u,v\right)\in E,E^{\prime }}\frac{d_{u}\Delta_{v}+d_{v}\Delta_{u}}{\Delta |E|d_{u}^{2}d_{v}^{2}}.$$

The temperature measures fluctuations in the internal structure of the time-evolving network and depends on two properties of the network. The first is the overall or global change of the number of edges Δ|E|, while the second property is a local one, which measures the change in degree for pairs of nodes connected by edges, i.e., $d_{u}\Delta_{v}+d_{v}\Delta_{u}$. Both quantities measure fluctuations in network structure, but at different levels of detail. The temperature is greatest when there are significant differences in the global number of edges and smallest when there are large local variations in edge structure, which do not result in an overall change in the number of edges.

Turning our attention in more detail to the term $d_{u}\Delta_{v}+d_{v}\Delta_{u}$ appearing in the numerator of the inverse temperature, it clearly measures the correlations between the degree of a node at one end of an edge and the change in degree at the other. When the correlation is large, then the reciprocal of the temperature is large, i.e., the temperature is low. On the other hand, low correlation corresponds to high temperature. Therefore, at low temperature, we can expect highly correlated changes in node degree, while at high temperature, these correlations are disrupted.

#### 2.3.2. Directed Edges

We can extend this analysis to directed graphs. According to Ye et al. [26], the approximate von Neumann entropy of a graph consisting entirely of directed edges, i.e., with no bidirectional edges, is:

(11) $$H_{D}=1-\frac{1}{\left|V\right|}-\frac{1}{{\left|V\right|}^{2}}\sum_{\left(u,v\right)\in E_{D}}\frac{d_{u}^{in}}{d_{u}^{out}}\cdot \frac{1}{d_{u}^{out}d_{v}^{in}}.$$

where $d_{u}^{in}$ is number of directed edges incident on node *u*, i.e., the in-degree of node *u*, $d_{u}^{out}$ is the number of edge exiting nodes *u*, i.e., the out-degree of node *u*, and $E_{D}$ the directed edge set of graph *G*. The edge commences at node *u* and ends at node *v*. It should be noted that the out-degree of the terminal node *v* does not participate in the expression for directed edge entropy. In terms of causality, this means it is determined by the causal past, but not the future of node *v*.

We can now repeat the incremental analysis for the directed version of the entropy. Considering only terms of first order in the change in in-degree and out-degree, we find:

(12) $$dH_{D}=H_{D}\left(G^{\prime }\right)-H_{D}\left(G\right)=\sum_{\left(u,v\right)\in E_{D}}\frac{d_{u}^{out}d_{v}^{in}\Delta_{u}^{in}-2d_{u}^{in}d_{v}^{in}\Delta_{u}^{out}-d_{u}^{in}d_{u}^{out}\Delta_{v}^{in}}{{\left(d_{u}^{out}\right)}^{3}{\left(d_{v}^{in}\right)}^{2}}$$

where $d_{u}^{in}$ is the in-degree at node *u*, $d_{u}^{out}$ the out-degree, and $\Delta_{u}^{in}$ and $\Delta_{u}^{out}$ the changes in in- and out-degree of node *u*. Again, the change in entropy takes the form of a correlation between the change in the in-degree or out-degree of a node and the product of the remaining two partial degrees. When the entropy change is substituted into the expression for reciprocal temperature, we again find that high correlation corresponds to low temperature.

### 2.4. Section Summary

In this section, we have detailed the development of the thermodynamic framework for network evolution analysis. In particular, we have employed three thermodynamic quantities, namely thermodynamic entropy, internal energy and temperature, to characterize the structure of time-varying complex networks. By analysing how these quantities change over time, we are able to track the time evolution of complex networks. It is also important to point out that one of the main advantages of the proposed framework is that these thermodynamic variables can be simply computed using graph statistics including graph size and node degree changes.

Another point worth noting is that when applying our approach to study real-world dynamic systems, it is critical to take into consideration how the corresponding dynamic network is built and, particularly, how the links connecting nodes in the network are established. For instance, according to Gorban et al. [27], given financial time series, the connections between financial entities can be assessed by correlations between either two individuals or two time moments. The two different measures, described as “varieties” and “volatilities”, respectively, have been shown to have different statistical properties, e.g., the latter does not require averaging in time when calculating correlation coefficients (locality), and thus could lead to different interpretations of our approach. A similar behaviour can be observed in the process of cell fate decision, as well [28].

## 3. Experiments and Evaluations

In this section, we test the performance of the proposed thermodynamic framework by applying it to analyse the time evolution of realistic complex networks. In particular, we aim to apply the thermodynamic variables, i.e., the entropy, energy, as well as temperature, to a few real-world time-varying networks in order to explore whether abrupt changes in structure or different stages in network evolution can be efficiently characterized.

The data we will analyse in the experiments are summarized as follows.

NYSE stock market network dataset: This is extracted from a database consisting of the daily prices of 3799 stocks traded on the New York Stock Exchange (NYSE). These data have been well analysed in [29], which has provided an empirical investigation studying the role of communities in the structure of the inferred NYSE stock market. The authors have also defined a community-based model to represent the topological variations of the market during financial crises. Here, we make use of a similar representation of the financial database. Specifically, we employ the correlation-based network to represent the structure of the stock market since many meaningful economic insights can be extracted from the stock correlation matrices [30,31,32]. To construct the dynamic network, 347 stocks that have historical data from January 1986–February 2011 are selected [29,33]. Then, we use a time window of 28 days and move this window along time to obtain a sequence (from Day 29–Day 6004) in which each temporal window contains a time series of the daily return stock values over a 28-day period. We represent trades between different stocks as a network. For each time window, we compute the cross-correlation coefficients between the time series for each pair of stocks and create connections between them if the maximum absolute value of the correlation coefficient is among the highest 5% of the total cross-correlation coefficients. This yields a time-varying stock market network with a fixed number of 347 nodes and varying edge structure for each of the 5976 trading days.

*Drosophila melanogaster* gene network dataset: This is extracted from DNA microarrays that contain the transcriptional profiles for nearly one-third of all predicted fruit fly (*Drosophila melanogaster*) genes through the complete life cycle, from fertilization to adult. The data are sampled at 66 sequential developmental time points. The fruit fly life cycle is divided into four stages, namely the embryonic (Samples 1–30), larval (Samples 31–40) and pupal (Samples 41–58) periods together with the first 30 days of adulthood (Samples 59–66). Early embryos are sampled hourly, and adults are sampled at multi-day intervals according to the speed of the morphological changes. At each time point, by comparing each experimental sample to a reference pooled mRNA sample, the relative abundance of each transcript can be measured, which can further be used as a gene’s expression level [34]. To represent this gene expression measurement data using a time-evolving network, the following steps are followed [35]. At each developmental point, the 588 genes that are known to play an important role in the development of the *Drosophila* are selected. These genes are the nodes of the network. The edges are established based on the distribution of the gene expression values, which can be modelled as a binary pair-wise Markov Random Field (MRF), whose parameter indicates the strength of undirected interactions between two genes. In other words, two genes are connected when their model parameter exceeds a threshold. This dataset thus yields a time-evolving *Drosophila* gene-regulatory network with a fixed number of 588 nodes, sampled at 66 developmental time points.

### 3.1. Thermodynamic Measures for Analysing Network Evolution

To evaluate how well our thermodynamic characterization method can be used to analyse the time evolution of complex systems, we thoroughly study the dynamic networks in both datasets. In particular, given the network structure at each time step, we compute the thermodynamic entropy together with internal energy according to Equations (7) and (8), respectively. Furthermore, we compute the temperature between networks at consecutive time steps using Equation (9). By investigating how these network thermodynamic variables evolve with time, it is interesting to see whether some critical events can be detected in the network evolution. These include financial crises or crashes in the stock market and the essential morphological transformations that occur in the development of the *Drosophila*.

#### 3.1.1. Financial Networks

**Figure 1:**
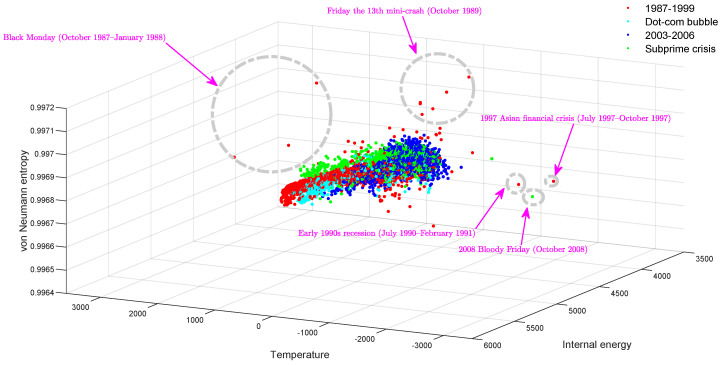
3D scatter plot of the dynamic stock correlation network in the thermodynamic space. Red dots: 1987–1999 data; cyan dots: Dot-com Bubble; blue dots: 2003–2006 background data; green dots: Subprime Crisis.

**Figure 2:**
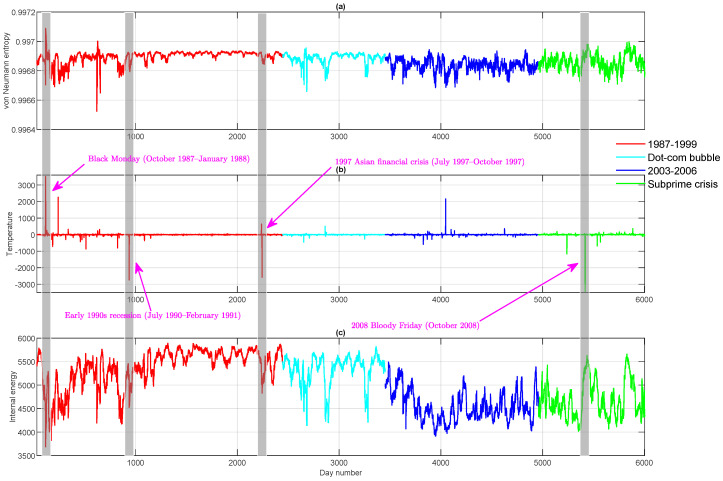
Top to bottom: (**a**) the von Neumann entropy versus time for the dynamic stock correlation network; (**b**) the temperature versus time for the dynamic stock correlation network; (**c**) the internal energy versus time for the dynamic stock correlation network.

In Figure 1, we show a three-dimensional scatter plot with each dimension representing a thermodynamic variable for the time-evolving stock correlation network. Essentially, such a plot represents a thermodynamic space spanned by entropy, internal energy and temperature. The most striking feature here is that the thermodynamic distribution of the time-evolving financial network shows a strong manifold structure with different phases of network evolution occupying different volumes of the thermodynamic space. More interestingly, the outliers, which indicate significant global events such as financial crises and stock market crashes, appear as peaks and troughs in the individual time series (see Figure 2). Examples include Black Monday, the 1997 Asian Financial Crisis and the 24 October 2008 stock market crash (Bloody Friday). Another interesting observation in Figure 1 is that the Dot-com Bubble period (approximately from 1999–2002), which is represented by cyan dots, is separated from the background data points and occupies a distinct region in the three-dimensional thermodynamic space. Theoretically, this is due to the fact that during the Dot-com Bubble period, a significant number of Internet-based companies were founded, leading to a rapid increase of both stock prices and market confidence. This considerably changed both the inter-relationships between stocks and the resulting structure of the entire market.

**Figure 3:**
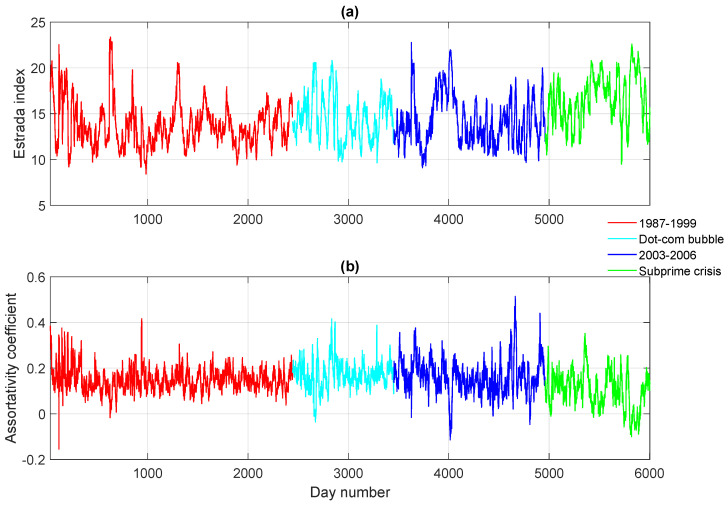
Top to bottom: (**a**) the Estrada index versus time for the dynamic stock correlation network; (**b**) the assortativity coefficient versus time for the dynamic stock correlation network.

To explore how our approach (especially the approximate von Neumann entropy) compares with existing graph characteristics in terms of revealing the network structural evolution across different phases, we pause here to investigate two well-known measurements for networks, namely: (1) the degree assortativity coefficient, originally developed by Newman [36]; and (2) the Estrada index [37]. Theoretically speaking, the main difference between the von Neumann entropy and the degree assortativity lies in that the former quantifies the network structural complexity, i.e., how far a given network deviates from a regular one, whereas the latter estimates the preference of nodes with different degrees being connected, although both their mathematical expressions contain the product of the degree of nodes that are linked in the network. On the other hand, the difference between the Estrada index and the von Neumann entropy is that the Estrada index exploits the spectrum of the network adjacency matrix instead of the Laplacian. The individual time series of the two measurements are reported in Figure 3. Clearly, both plots show significant fluctuations over the entire time period. Although critical events such as Black Monday and the 1997 Asian Financial Crisis appear to be peaks and troughs in the figure, they cannot be easily distinguished from a large number of other fluctuations. Moreover, compared with Figure 2, the time periods in which the network structure remains relatively stable cannot be identified, as both time series display continuous fluctuations. These interesting observations together suggest that, by viewing critical event networks as the outliers that deviate far from the regular network, which corresponds to the stable phase in the time evolution, our thermodynamic framework turns out to be a more appropriate option for analysing the structural changes of dynamic networks. This is because the von Neumann entropy can measure the distance between a given network and a regular one, which cannot be readily estimated by other existing network characteristics.

**Figure 4:**
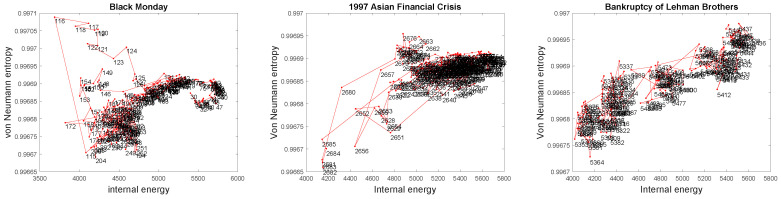
Trace of the time-evolving stock correlation network in the entropy-energy plane during financial crises (the number beside the data point is the day number in the time series). **Left**: Black Monday (from Days 30–300); **Middle**: Asian Financial Crisis (from Days 2500–2800); **Right**: Bankruptcy of Lehman Brothers (from days 5300–5500).

We now study three financial crises in detail and explore how the thermodynamic variables can be used to unravel how the stock market network structure changes with time. In Figure 4, we show the trace of the stock network on the entropy-energy plane during Black Monday (left panel), the Asian Financial Crisis (middle panel) and the Lehman Brothers Bankruptcy (right panel), respectively. The number beside each data point represents the day number in the time series. From the figure, before Black Monday, the network structure remains relatively stable; neither the network entropy nor the internal energy changes significantly. However, when Black Monday takes place (Days 115 and 116 in the time series), the network undergoes a considerable change in structure since the entropy increases dramatically. Then, the network entropy slowly decreases after the stock market crash, which implies that the stock correlation network gradually returns to its normal state (before crisis). A similar pattern can be observed concerning the 1997 Asian Financial Crisis, which is shown in the middle panel, as well. In short, the stock market undergoes a significant crash in which the network structure undergoes a significant change, as signalled by a large drop in network entropy. The crash is followed by a slow recovery. It is interesting to note that for the Lehman Brothers Bankruptcy case, as the time series evolves, both the network entropy and the internal energy continue to grow gradually, which yields a very different pattern as compared to previous cases. Therefore, the difference in the network structure behaviour during different financial crises implies that our thermodynamic representation can be used to both characterize and distinguish between different critical events in the network evolution.

**Figure 5:**
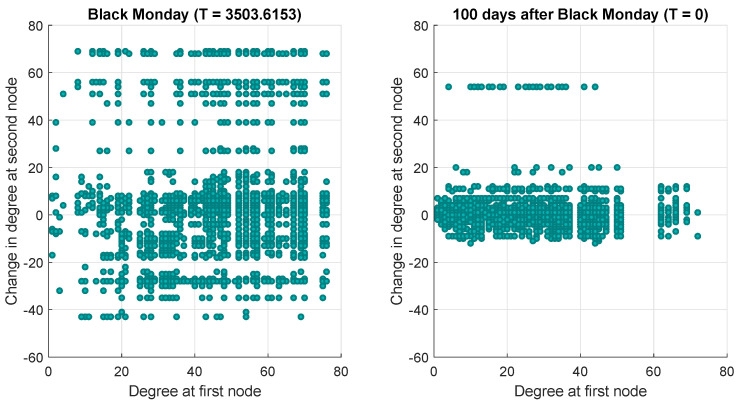
Scatter plots of $\Delta_{v}$ versus $d_{u}$ for high and low temperature networks.

**Figure 6:**
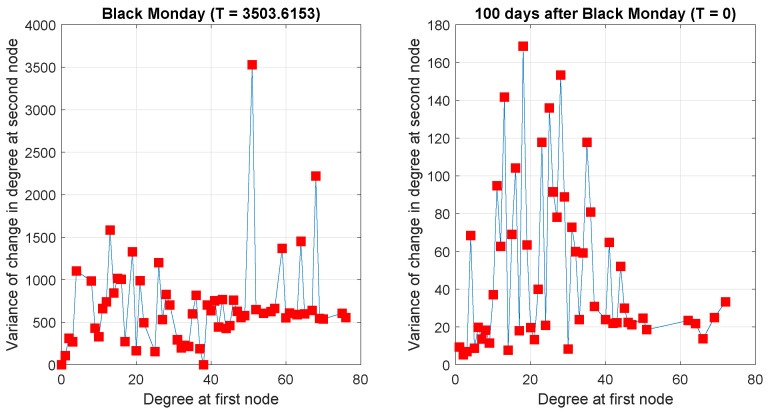
Scatter plots of variance of $\Delta_{v}$ versus $d_{u}$ for high and low temperature networks.

Next, we particularly concentrate on the temperature variable, which measures the structural difference of networks at consecutive time steps. From the definition of temperature Equation (10), clearly the temperature depends on changes in node degree. Mathematically, for an undirected graph, the reciprocal of the temperature is determined by the quantity $d_{u}\Delta_{v}+d_{v}\Delta_{u}$. Therefore, in order to investigate the correlation between node degree and node degree change, we show a scatter plot of $\Delta_{v}$ versus $d_{u}$ for nodes *u* and *v* connected by an edge in Figure 5. We consider two pairs of consecutive networks, respectively: the first contains networks in the proximity of the Black Monday epoch (left panel), whereas the second consists of networks far away from it (right panel). The main difference between the two sub-plots lies in that for the case of the Black Monday networks, there is no correlation between $d_{u}$ and $\Delta_{v}$, while in the case of the second pair, there is a regression line of approximately zero slope. The temperature between networks in the former pair is particularly high, whereas the latter corresponds to a very low temperature. Another feature to note from the two plots is that for a given degree, the variance of the degree changes is greatest at high temperature. To illustrate this point, Figure 6 shows the variance of the degree change as a function of degree. In the case of the Black Monday networks, the variance is much larger than in the case of the second network pair, far away from it.

#### 3.1.2. Gene Regulatory Network

**Figure 7:**
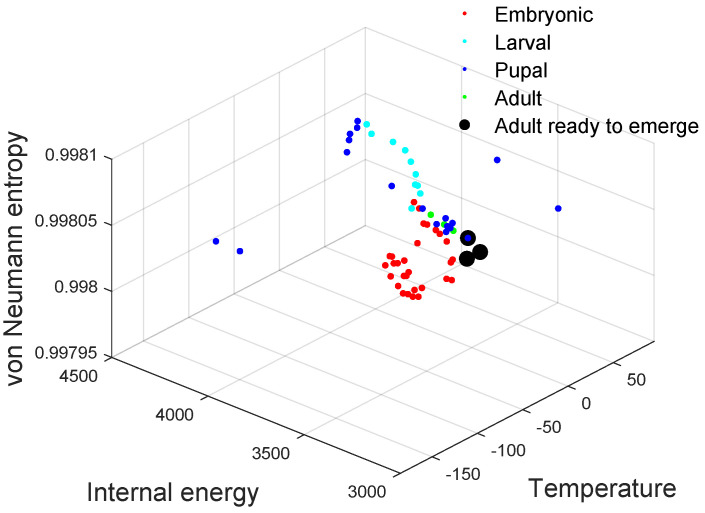
3D scatter plot of the *Drosophila melanogaster* gene regulatory network in the thermodynamic space. Red dots: embryonic period; cyan dots: larval period; blue dots: pupal period: green dots: adulthood; black dot: adult ready to emerge.

**Figure 8:**
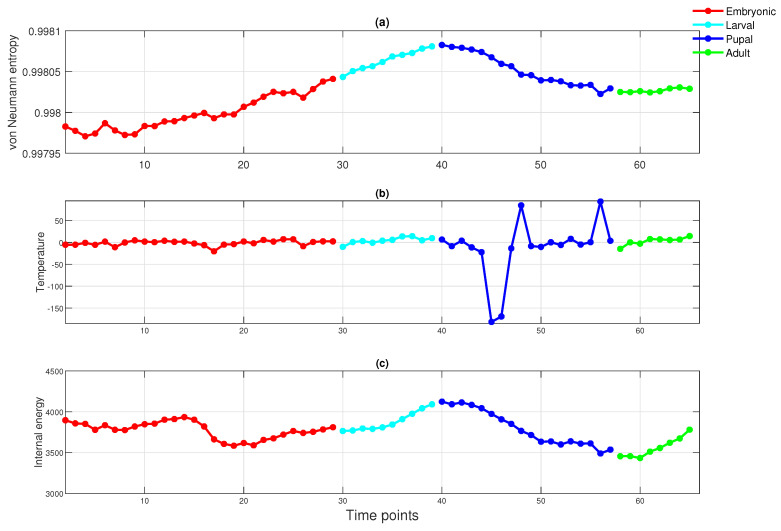
Top to bottom: (**a**) the von Neumann entropy versus time for the *Drosophila melanogaster* gene regulatory network; (**b**) the temperature versus time for the *Drosophila melanogaster* gene regulatory network; (**c**) the internal energy versus time for the *Drosophila melanogaster* gene regulatory network.

We now apply the thermodynamic framework to the fruit fly network, i.e., the *Drosophila* gene regulatory network in the second dataset. Similar to the experiments performed on the financial data, we again show the three-dimensional scatter plot of the thermodynamic variables of the time-varying network in the thermodynamic space (Figure 7), together with the entropy, energy and temperature times series (Figure 8). The four developmental stages are shown in different colours. Some key observations can be made. First, the different stages of evolution are easily distinguished by the thermodynamic variables. For instance, from Figure 8, due to the early development of an embryo, the red curve (embryonic period) shows some fluctuations. This is attributable to strong and rapidly changing gene interactions, because of the need for rapid development. Secondly, in Figure 7, the pupal stage data points are relatively sparsely distributed in the thermodynamic space. This is attributable to the fact that during this period, the pupa undergoes a number of significant pupal-adult transformations. Moreover, as the organism evolves into an adult, the gene interactions that control its growth begin to slow down. Hence, the green points (adulthood) remain stable. Finally, the black data points are well separated from the remainder of the developmental samples and correspond to the time when the adult emerges.

To summarize, in this section, we have implemented computational experiments on two realistic time-evolving complex systems extracted from financial and biological domains, respectively. For the stock market data, we have particularly analysed a few well-known stock market crashes and have demonstrated that the thermodynamic entropy, internal energy together with temperature provide a powerful tool for detecting abrupt events and characterizing different stages in the network evolution. The same conclusion can also be drawn based on the results of the fruit fly life cycle network analysis.

## 4. Conclusions

It is of fundamental importance to have methods in hand to characterize and understand the time evolution of time-varying complex systems. To tackle this particular problem, in this paper, we have developed a few global variables for networks, namely the thermodynamic entropy, internal energy and temperature, and have united them as a whole to analyse the structural properties of time-evolving networks. In other words, we have adopted a thermodynamic framework to visualise and understand the network evolution. Specifically, based on statistical thermodynamics, this method starts with a recently derived expression for the von Neumann entropy of a network. The method then connects the microscopic configurations of a network with the normalized Laplacian eigenstates. In this way, we have shown that the von Neumann entropy can be interpreted as the thermodynamic entropy of a network. The method further defines the network internal energy, which is determined by the number of edges in the network. Finally, the thermodynamic temperature is a measure that gauges the structural fluctuations between networks at consecutive time points, via changes in the number of edges and individual node degree changes.

To demonstrate that the proposed framework serves as a powerful tool for detecting critical events and distinct periods in the time evolution of real-world complex systems, we have evaluated the method experimentally using data taken from the financial and biological domains. The experimental results have confirmed that the thermodynamic variables together provide an efficient framework for analysing the evolutionary properties of dynamic networks.

In the future, in order to improve the thermodynamic characterizations so that they can become more effective in identifying critical events and significant time stages in the evolution of time-varying networks, we could turn our attention to the quantum physics. In particular, we would be interested in exploring whether partition functions from different quantum statistics, such as the Bose–Einstein partition function and the Fermi–Dirac partition function, can be used for the purpose of providing a more efficient way to probe dynamic network structure.
